# Comprehensive Genomic and Epigenomic Analyses on Transcriptomic Regulation in Stomach Adenocarcinoma

**DOI:** 10.3389/fgene.2021.778095

**Published:** 2022-02-11

**Authors:** Junxing Chen, Weinan Liu, Jiabin Du, Pengcheng Wang, Jintian Wang, Kai Ye

**Affiliations:** Department of Oncology Surgery, Second Affiliated Hospital of Fujian Medical University, Quanzhou, China

**Keywords:** stomach adenocarcinoma, integrative analysis of genomic and epigenomic regulation, DNA copy number–correlated genes, methylation-correlated genes, mutation—genetics

## Abstract

**Background:** DNA methylation (MET)–mediated transcriptomic disturbance and copy number variations (CNVs) exert a significant influence in stimulating the heterogeneous progression of stomach adenocarcinoma (STAD). Nevertheless, the relation of DNA MET with CNVs, together with its impact on tumor occurrence, is still unclear.

**Methods:** The messenger RNA (mRNA) expression (EXP) profiles, DNA MET, and DNA copy numbers, together with STAD mutation data, were collected from the TCGA official data portal. We employed circular binary segmentation algorithm in “DNAcopy.” library of R package for mapping DNA CNV data at genetic level for all samples based on the segmented CNV data. Stable clusters of samples were recognized using negative matrix factorization cluster analysis based on 50 iterations and the “brunet” method using the MET-correlated (METcor) and CNV-correlated (CNVcor) genes. The R package “iCluster” method was utilized to comprehensively analyze the EXP, MET, and DNA CNV profiles.

**Results:** A total of 313 STAD samples were isolated for checking DNA copy numbers and MET and for measuring EXP. In accordance with our results, we discovered obvious co-regulation of CNVcor genes and METcor counterparts. Apart from that, these genes were subject to multi-omics integration. Meanwhile, three subtypes of STAD were detected and confirmed based on independent data. Among them, the subtype with increased aggressiveness was related to decreased mutation frequencies of ARID1A, PIK3CA, ZFHX3, SPECC1, OBSCN, KMT2D, FSIP2, ZBTB20, TTN, and RANBP2, together with the abnormal levels of JPH3, KCNB1, and PLCXD3.

**Conclusion:** According to the results, these aforementioned genes exerted crucial roles in the development of invasive STAD. Our findings on transcriptomic regulation genomically and epigenetically facilitate the understanding of the STAD pathology from different aspects, which help to develop efficient anti-STAD therapy.

## Background

Gastric cancer (GC), the cancer with high malignancy rate, accounts for about 7.7% of cancer-related deaths in the world in 2020. Related treatments have been developed for GC. However, it is still the fatal malignant tumor that ranks the fourth place in terms of its mortality globally, which is ascribed to its advanced diagnosis and high morbidity (Sung et al., 2021). Typically, the overall survival (OS) rate of GC at 5 years is less than 25%, especially for the recurrent and advanced types (Bray et al., 2018). Stomach adenocarcinoma (STAD) takes up about 90% of global GC cases (Bu and Ji, 2013; Karimi et al., 2014). Most STAD patients among western countries are diagnosed at the metastatic or advanced stage (Van Cutsem et al., 2011). Although great advances have been attained in chemotherapy, radiotherapy, and surgery, prognosis of STAD still remains dismal. Some GC patients present diverse prognostic outcomes and therapeutic responses, even though they are at the same TNM (Tumor Node Metastasis) stage (Shiratsu et al., 2014). Early STAD diagnosis markedly enhances patient outcomes. As a result, the biomarkers for diagnosis and prognosis are in urgent need to improve the STAD diagnosis and to predict the outcomes for patients.

Markers for diagnosis and prognosis can be used to closely monitor and treat the high-risk patients, so as to extend their OS time. The common STAD biomarkers applied in clinic are mainly the clinicopathological parameters, such as tumor stage, age, chemotherapy response, and infection with *Helicobacter pylori* (Ye et al., 2004). Developing new biomarkers can introduce and design new therapeutic strategies for improving the survival for patients. Therefore, comprehensive examination of the disease molecular characteristics is of crucial necessity.

In recent years, the huge multi-omics greatly contributes to comprehensively displaying disease dysregulation at genomic and epigenetic levels (Woo et al., 2017). Genomic alternations induced by cancer filing, such as DNA mutations and copy number variations (CNVs), are common during tumor genesis, which promote cancer progression (Shi et al., 2016). In addition, cancer genomic regulation *via* DNA methylation (MET) at an epigenetic level exerts a crucial part in the behaviors of various cancers, including STAD (Peng et al., 2020; Song et al., 2020). Genomic profiling research suggests that genomic and epigenomic dysregulation is highly heterogeneous. DNA CNVs exert a vital part in STAD for regulating STAD development; in addition, the resultant transcriptional dysregulation is potentially a driving event during the progression of STAD (Feng et al., 2019; Cai et al., 2020). Besides, research on DNA MET profiling suggests that epigenetic regulation is highly significant in the development of cancer from the points of view of biology and clinic (Choi et al., 2017; Ebrahimi et al., 2020). At the same time, some critical tumor-associated genes, such as RASSF1A, SAMD14, and SOCS3, can modulate DNA MET, thereby regulating their functions (Balgkouranidou et al., 2015; Han et al., 2020; Xu et al., 2020).

**FIGURE 1:**
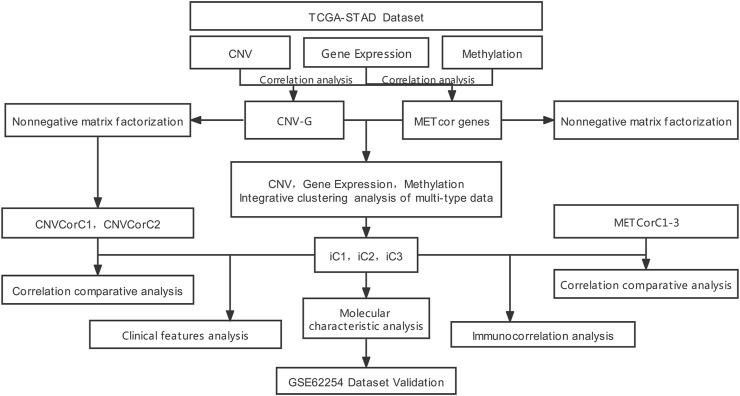
The work flow chart of this study.

Nonetheless, the association between DNA MET and CNVs is still unknown, even though DNA MET alternations and CNVs affect the whole genome of cancer. Besides, the impact of this association during cancer development remains unclear. In the current work (flowchart is shown in Figure 1), we extracted STAD samples for analyzing DNA copy numbers and MET, together with the messenger RNA (mRNA, EXP) expression. In addition, genes related to DNA copy numbers (CNVcor) and MET (METcor) were identified according to related gene levels in the aforementioned samples, separately, so as to recognize genes with genomically and/or epigenetically modulated expression. Of them, CNVcor genes suggested transcriptional dysregulation according to DNA copy numbers, while METcor ones represented transcriptional dysregulation according to DNA MET. CNVcor gene levels were evidently related to METcor gene levels, which indicated cancer transcriptomic co-regulation via genomic DNA CNVs and epigenetic DNA MET abnormalities. Besides, we conducted multi-omics integration of METcor and CNVcor genes, so as to examine the typical molecular subtypes to predict the prognosis of STAD. Furthermore, we also supplied the differential and correlation analyses on tumor microenvironment and immune infiltration among different subtypes and different samples, which have improved the guiding significance for the individualized immunotherapy for GC patients. Novel specific targets and biomarkers to distinguish different subtypes of cancer were identified through intensive systematic analysis.

## Methods

### mRNA Expression Patterns, DNA MET, and DNA Copy Numbers

A STAD dataset was downloaded from TCGA for analyzing related results. The mRNA expression profiles, DNA MET, and DNA copy numbers, together with STAD mutation data, were collected from the TCGA official data portal. Each sample mark was matched in every platform; afterwards, altogether 313 datasets were utilized (Supplementary Table S1), which had corresponding datasets for mRNA expression profiles, DNA MET, and DNA copy numbers. In addition, gene expression patterns were standardized through log2 transformation and quantile normalization, followed by HUGO official symbol aggregation. The expression profiles were subsequently standardized through eliminating the average values from each probe within non-cancer tissues, which stood for the fold change in tumor samples relative to healthy samples. Thereafter, we utilized the R package circular binary segmentation algorithm in “DNAcopy.” library for mapping DNA CNV data at the genetic level for all samples based on the segmented CNV data (Zhang et al., 2017). With regard to the DNA MET profiles, the β-values of the probe were filtered for eliminating probes positioned on the sex chromosomes. Later, probes located in the regions associated with the CpG islands were mapped to associated genes, such as Shore regions, CpG islands, Shelf, differentially methylated regions, first-exon regions, 5′UTR, and the gene promoter areas that contained 2,500 upstream basic groups from TSS. We eliminated probes that had over 30% missing values among the samples from every processed pattern.

The “liftOver,” library of R package was utilized for every dataset, so as to restore the genomic coordinates of probe against human reference genome hg38. Afterwards, we matched the probes to related ones from EXP profiles. Cancer-specific alterations were computed through eliminating the mean probe intensity within non-cancer tissues. Then, probes on the sex chromosomes and those that had over 50% missing values were eliminated, and related information was input with the sk-nearest neighbor algorithm. Thereafter, we calculated the pairwise Pearson’s correlation coefficients of all genes across the matched EXP and CNV profiles, as well as EXP and MET, respectively. If over one probe was mapped to one gene, the probe that had the average or the smallest correlation coefficient was utilized to be the typical pair-matched probes of MET and CNV profiles, separately.

### Cluster Analysis on the Genomic Patterns at Different Levels

Stable clusters of samples were recognized by cluster analysis of negative matrix factorization (NMF) based on 50 iterations and the “brunet” method through CNVcor and METcor genes, separately (Zeng et al., 2019). Notably, we set k as 2–10 and calculated the best k according to the observed consensus map, together with the cluster cophenetic correlation. At the same time, to examine consensus membership matrix, the mean silhouette width was computed by “NMF.” of R package. Regarding all membranes, we set the lowest k as 10. Then, we utilized R package “iCluster” method for comprehensively analyzing the EXP, MET, and DNA CNV profiles; besides, 20 iterations and the default parameters were utilized (Shen et al., 2012).

### Evaluation and Identification of Immune Cell Infiltration

In this study, we identified and evaluated the abundance of immune infiltrates by the TIMER algorithm, which is a resource for systematic and extensive analysis of immune infiltrates (totally six cell types, namely: B cells, CD4^+^ T cells, CD8^+^ T cells, neutrophils, macrophages, and dendritic cells) across diverse cancer types.

## Results

### Transcriptomic Alternations in DNA Copy Number or DNA MET

DNA CNVs and MET at genetic and epigenomic levels, as well as gene EXP patterns, were collected from 313 STAD specimens. Then, the raw materials were preprocessed according to the method described previously in “Methods”. Later, the correlation coefficients of DNA CNV or MET profiles with corresponding mRNA EXP data were computed, so as to evaluate the influence of epigenomic and/or genomic abnormalities. Thereafter, we standardized the correlation coefficient r according to Fisher’s Z-transformation for variance stabilization.

**FIGURE 2:**
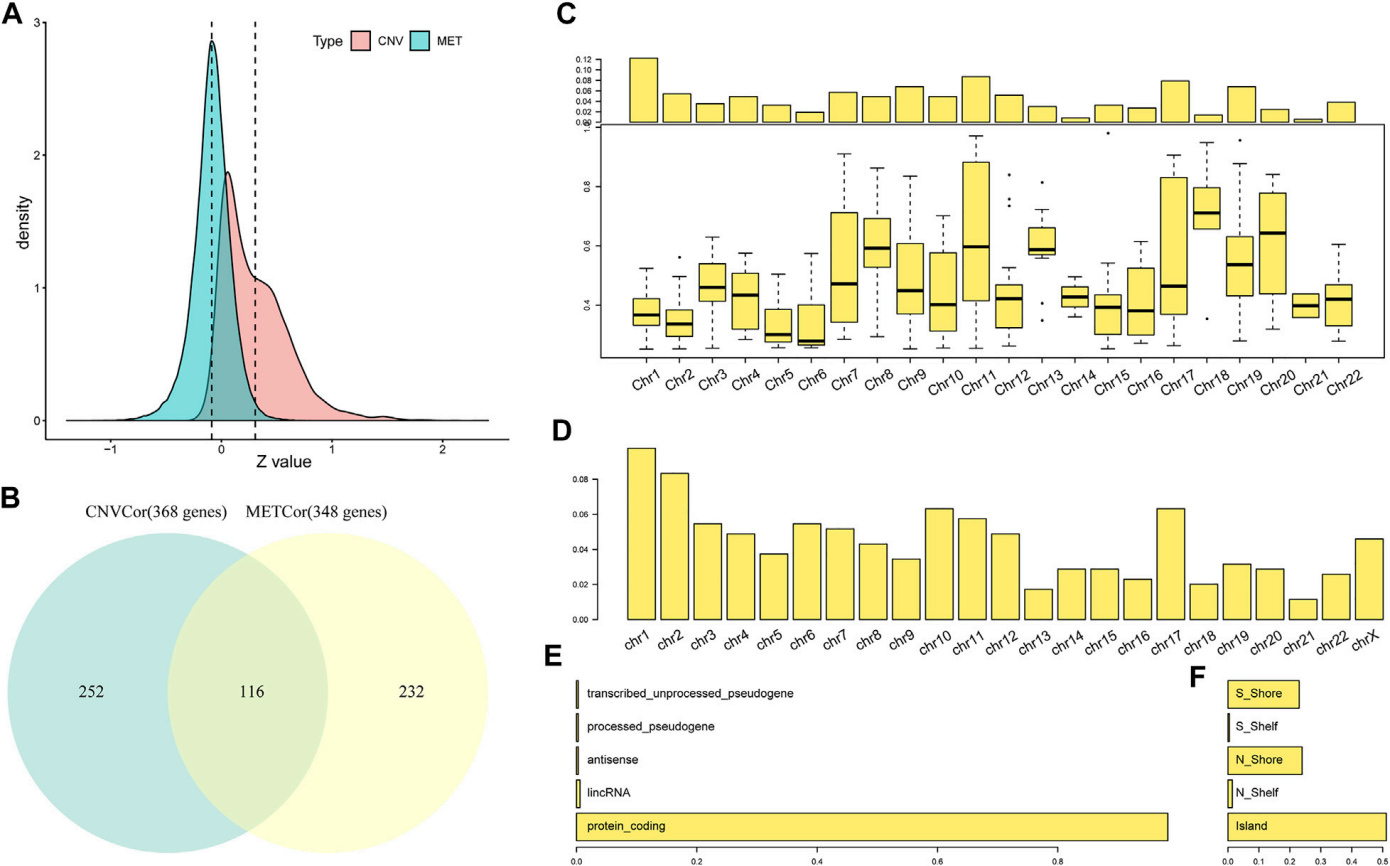
Recognition of DNA methylation–correlated (METcor) and DNA copy number–correlated (CNVcor) genes of STAD. **(A)** Correlation coefficient distribution of mRNA expression with DNA MET or DNA copy numbers among different specimens is presented, respectively. **(B)** The Venn diagram displays the METcor and CNVcor gene numbers. Overlapped gene number between METcor and CNVcor genes is indicated. **(C)** Proportions of CNVcor genes in the total number of genes in every chromosome arm. **(D)** METcor gene proportions in the overall number of genes in every chromosome arm. **(E,F)** Genomic locations of DNA MET probes were classified according to the relationships with genes (left) and CpG islands (right) in positions, separately.

Consistent with a prior study, correlation coefficient distribution of DNA CNV with related EXP data markedly skewed to the right (skewness = 1.2352, *p* < 1e^−5^). In comparison, the correlation coefficients of DNA MET with related EXP data skewed to the left (skewness = −0.37363, *p* < 1e^−5^) (Figure 2A), which indicated that DNA CNVs and MET abnormalities positively and negatively modulated transcription, respectively.

A great number of genes participated in the two aforementioned gene sets (CNVcor and METcor genes, Supplementary Tables S2, S3); as a result, we selected genes that were markedly correlated with OS for later analyses (log rank *p* < 0.05). Then, we screened gene signatures showing positive correlation to examine DNA copy number (CNVcor, *n* = 368) and those showing negative correlation for DNA MET (METcor, *n* = 348). CNVcor genes indicated that transcriptional dysregulation was dependent on DNA CNVs, but METcor genes suggested that it was dependent on MET. CNVcor genes were not overlapped with METcor ones; of them, 116 overlapped genes were identified, which suggested that CNVcor and METcor genes specifically regulated transcriptional dysregulation (Figure 2B).

CNVcor genes preferred DNA CNVs within some genomic regions, especially on chromosomes 10, 17, 18, and 21 (Figure 2C and Supplementary Table S4). Consistent with prior research, CNVcor genes were abundant on chromosomes 10 and 17, which suggested that gene expression was sensitive to DNA level in certain regions (Arakawa et al., 2017). In addition, METcor genes were found within desired chromosomal regions, like chromosome 19 (Figure 2D and Supplementary Table S5), most of which were genes that encoded proteins (Figure 2E) and were distributed in the CpG islands (Figure 2F). This study suggested that CNVcor and METcor genes were remarkably effective on transcriptional dysregulation of STAD, which required to be further examined in future studies.

### Different CNVcor and METcor Gene–Dependent Molecular Subtypes

**FIGURE 3:**
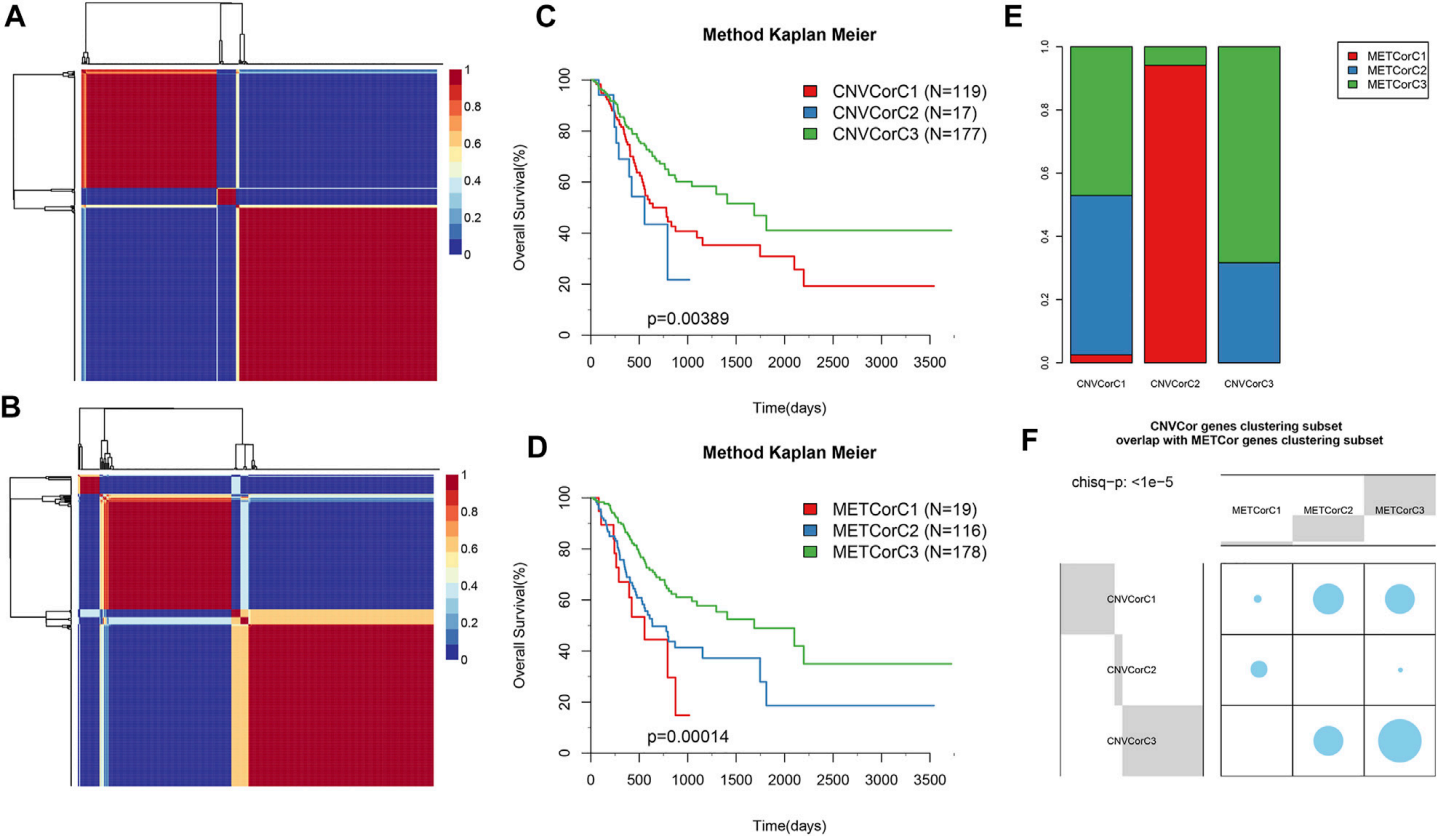
Identification of STAD molecular subtypes by METcor and CNVcor genes. **(A,B)** Plots displaying cluster results of NMF of CNVcor gene from CNV data **(A)** and METcor genes from MET data **(B)**, separately. **(C,D)** OS Kaplan–Meier curves for subtypes stratified based on NMF cluster analysis on CNVcor **(C)** and METcor **(D)** genes, separately. **(E,F)** Subtypes identified according to CNVcor show marked overlaps with those identified by METcor.

Subsequently, the effects of CNVcor and METcor gene expression on predicting prognostic subgroups were explored. NMF cluster analysis was performed for all gene set data, and we set k as 2–10; later, k value was calculated for each profile (k = 3 for CNV and MET, respectively) (Figures 3A,B). Surprisingly, CNVcor gene–identified subtypes were remarkably overlapped with the METcor gene–identified ones (*p* < 1e^−5^ upon χ^2^ test), and such results were consistent with CNVcor and METcor gene regulation in STAD (Figures 3E,F). In addition, Kaplan–Meier (KM) curve results suggested that CNVcor or METcor gene–identified subtypes predicted the OS of patients (Figures 3C,D), separately (*p* < 0.05).

Molecular subtypes associated with CNVcor and METcor gene expression were identified at various aspects. iCluster, the integrated cluster method, was employed to integrate genomic data on mRNA EXP, DNA CNVs, and MET. Afterwards, cluster analysis was carried out with the cluster number k of 2–4. Altogether, 20 cluster iterations at K = 4 (category 5), K = 3 (category 4), and K = 2 (category 3) were carried out separately to assess the best iCluster cluster results. According to our findings, stable cluster results were obtained at K = 2 relative to those at K = 3 or 4 (Supplementary Figures S1, S2). As a result, all samples were clustered into three subclasses of iC1–iC3 (*n* = 93, 100, and 120, respectively). Supplementary Figures S3A,B display the cluster results of those three subclasses, and Supplementary Table S6 presents those of all samples.

**FIGURE 4:**
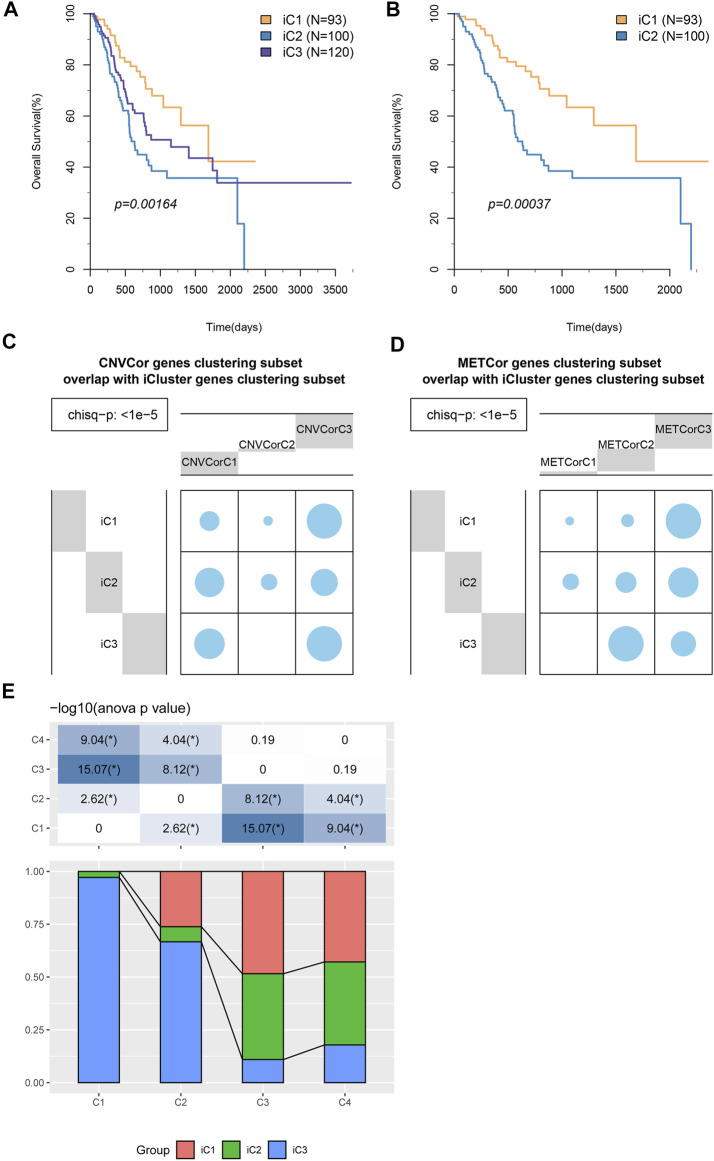
STAD molecular subtypes identified through iCluster analysis. **(A)** OS Kaplan–Meier curves for subtypes stratified based on iCluster (iC1–iC3). **(B)** OS Kaplan–Meier curves for iC1 and iC2. **(C,D)** Subtypes identified based on iCluster analysis showed evident overlaps with CNVcor **(C)** and METcor **(D)** gene–identified subtypes. **(E)** The distribution of samples of iC1–3 subtypes in four molecular subtypes of TCGA database.

KM analysis results suggested that iC1 attained the optimal OS across those three subtypes (*p* < 0.05, Figure 4A). The OS of patients in iC1 subgroup was compared with that of the other two subgroups (Figure 4B and Supplementary Figure S4), and the results suggested that the difference in prognosis between iC1 and iC2 subgroups was statistically significant (*p* < 0.001). Notably, those icluster-identified subtypes remarkably overlapped with CNVcor and METcor gene–identified counterparts (*p* < 1e^−5^, χ^2^ test, Figures 4C,D). In addition, we have explored the distribution of samples of iC1-3 subtypes in four molecular subtypes of TCGA database, and the results (Figure 4E) suggested that samples of iC3 subtype were mainly distributed into C1 and C2 subtypes, while samples of iC2 subtype were mainly distributed in C3 and C4 subtypes. According to the aforementioned findings, comprehensive analysis on CNVcor and METcor genes facilitated to detect various molecular subtypes, and all of them showed heterogeneous combinations of genomic and epigenomic features associated with prognosis and transcriptional dysregulation.

### Comparisons of DNA CNV and MET Abnormalities

**FIGURE 5:**
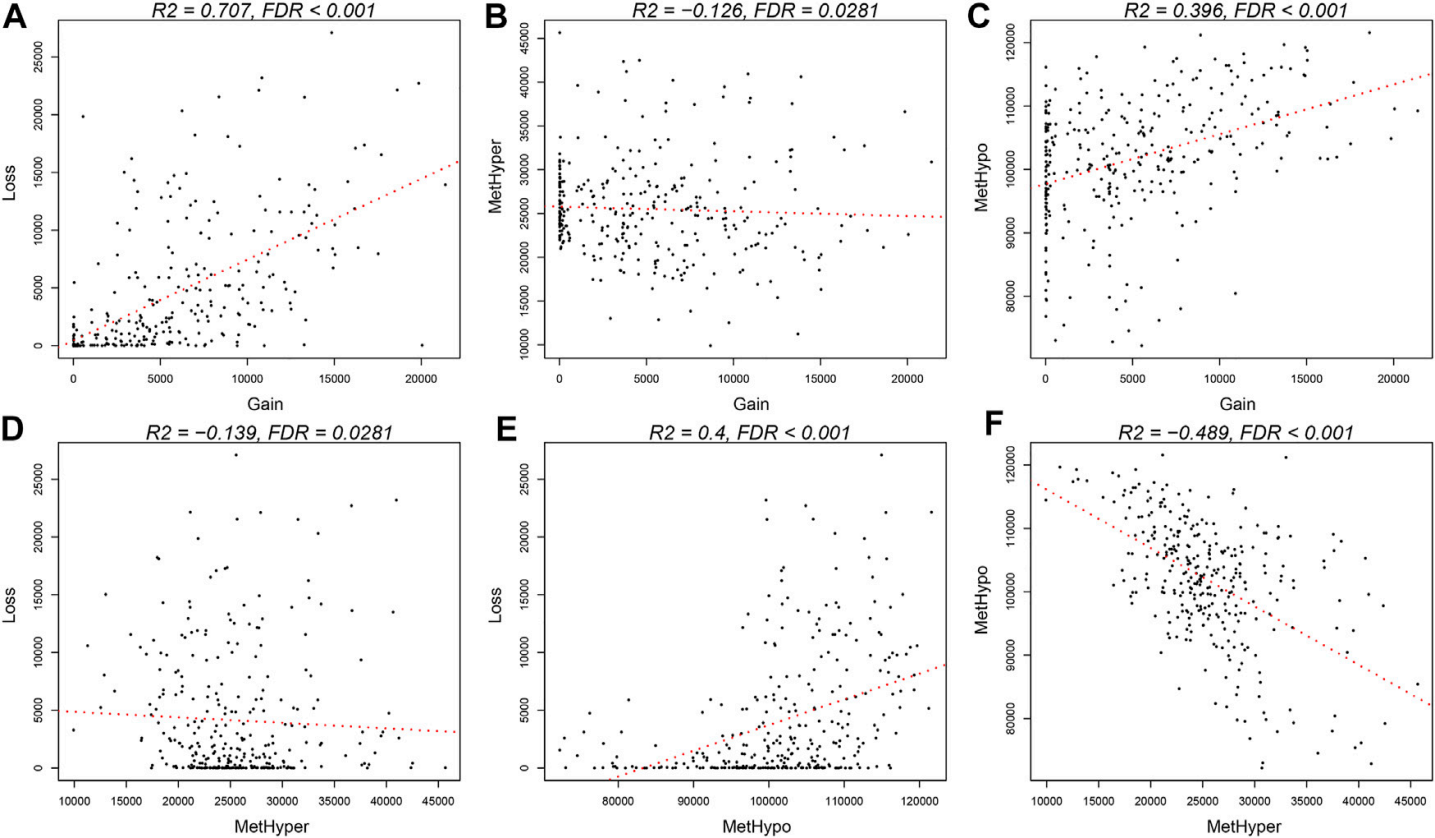
Coordinated DNA MET abnormalities and DNA CNVs in STAD. **(A,F)** DNA CNV or DNA MET abnormalities were identified at the threshold fold change of >0.2 relative to the means in normal tissues, respectively. Directional DNA CNV-gain (CNVgain) and CNV-loss (CNVloss), together with DNA hypomethylation (METhypo) and hypermethylation (METhyper), in all samples are presented, respectively. **(B–E)** Plots display pairwise occurrence rates of CNVloss, CNVgain, METhypo, and METhyper genes in each sample.

The frequencies between genome-wide DNA MET abnormalities and DNA CNVs were compared after batch effect correction. In addition, DNA copy-number gain (CNVgain, *β* > 0.3) and loss (CNVloss, *β* < −0.3), together with DNA hypermethylation (METhyper, *β* > 0.8) and hypomethylation (METhypo, *β* < 0.2), were computed according to the determined threshold (fold change of 0.3), which were subsequently compared with the level of every probe. Our results indicated that (Supplementary Table S7) CNVgain frequency showed marked correlation with CNVloss frequency (*p* < 1e^−5^, Figure 5A). Besides, METhyper frequency displayed evident correlation with METhypo frequency (*p* < 1e^−5^, Figure 5F). Directional METhyper and CNVgain were tightly correlated with CNVloss, which indicated that all correlations were directional abnormality free (Figures 5B–E, *p* < 0.05). In conclusion, our results suggested that STAD patients with increased frequency of DNA CNVs had elevated frequency of DNA MET abnormality. Correlations between the frequency of abnormal METcor and CNVcor genes indicated the close correlation of DNA MET with DNA CNVs.

### Identification of STAD Subtype Key Features

**FIGURE 6:**
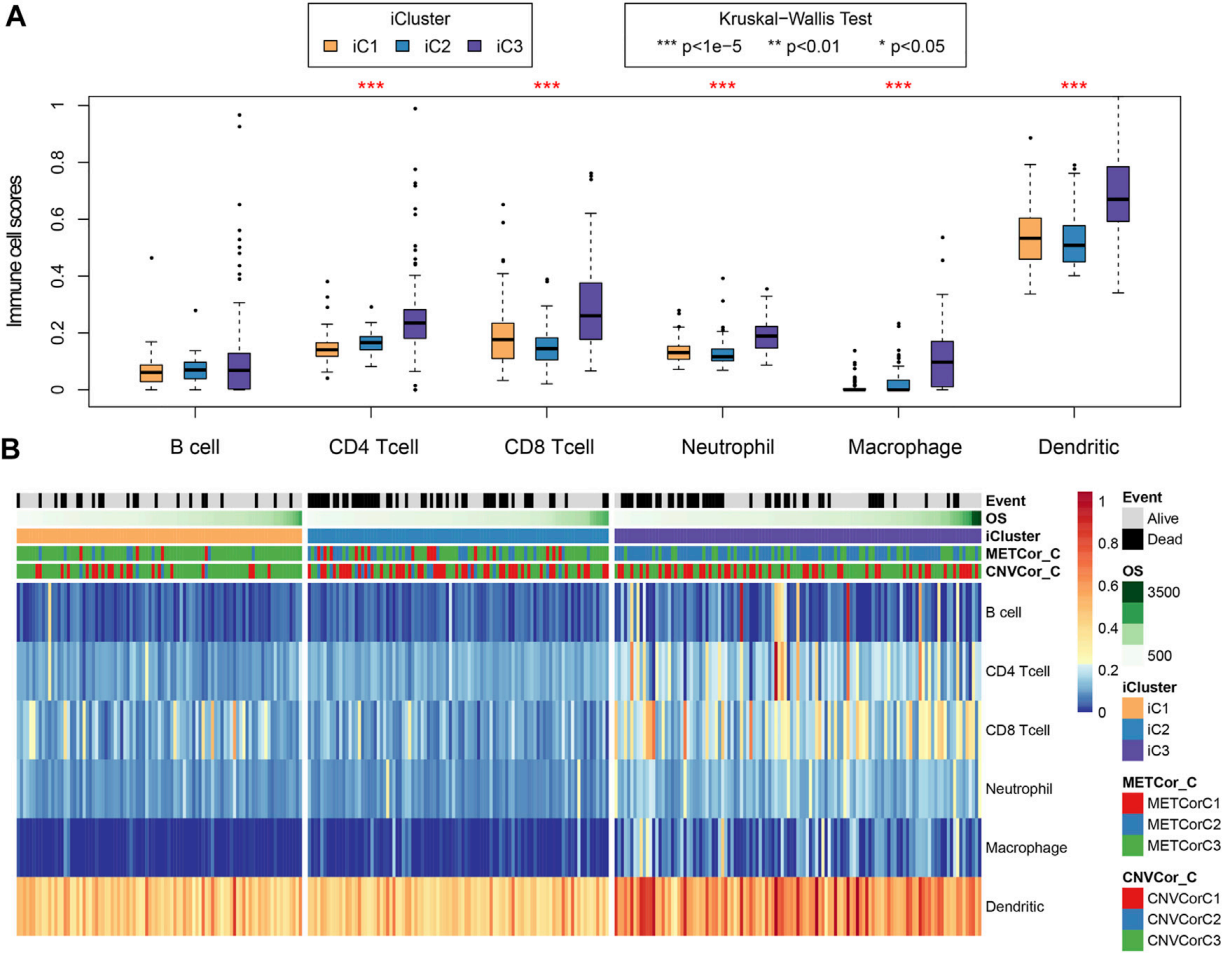
Key STAD immune features. Sample immune scores among the three subtypes were computed and analyzed by the tumor immune estimation resource (TIMER) method. Scores of six immune cells in all samples were determined **(B)** and analyzed compared with those in samples of other subtypes **(A)**.

First, clinical features (including TNM, stage, gender, and primary site) were compared across those three subtypes. Supplementary Figure S5 and Table 1 indicated that the differences in clinical characteristics were not statistically significant among three STAD subtypes. Nonetheless, as for stage distribution, III and IV samples mainly belonged to iC2 subtype that had the poorest prognosis. In addition, considering the significance of TCGA to study the infiltration and the complex interaction of immune cells within tumor microenvironment (TME) (El-Arabey et al., 2020), tumor immune microenvironment (TIME) status of samples across these three subtypes were also calculated and determined according to the tumor immune estimation resource (TIMER) method (Li et al., 2016) (Supplementary Table S8) (Li et al., 2016). As observed, differences in the 5/6 immune cell scores [CD4^+^ T, CD8^+^ T, macrophages, dendritic cells (DCs), neutrophils] of samples across these three subtypes were statistically significant (Figures 6A,B, *p* < 1e^−5^). The aforementioned results indicated that immunocyte infiltration degree or the immune microenvironment of STAD was correlated with DNA CNV or MET level.

**TABLE 1:** Clinical information of STAD patients across the three subtypes were compared

Event	Total	iC1	iC2	iC3
Alive	188	69	47	68
Dead	125	22	49	49
T				
T1	14	7	5	2
T2	63	17	26	20
T3	146	41	47	58
T4	81	26	18	37
N				
N0	90	35	24	31
N1	80	20	25	35
N2	65	20	22	23
N3	62	14	23	25
NX	7	2	2	3
M				
M0	275	87	86	102
M1	18	2	5	11
MX	11	2	5	4
Stage				
I	39	14	14	11
II	99	34	27	38
III	129	36	40	53
IV	28	3	13	12
Un	9	4	2	3
Grade				
G1	8	2	3	3
G2	107	37	49	21
G3	180	51	40	89
GX	9	1	4	4
Age (years)				
0–50	27	9	7	11
50–60	72	15	20	37
60–70	91	23	33	35
70–80	99	38	32	29
80–100	15	6	4	5
Gender				
Female	101	29	27	45
Male	203	62	69	72

**FIGURE 7:**
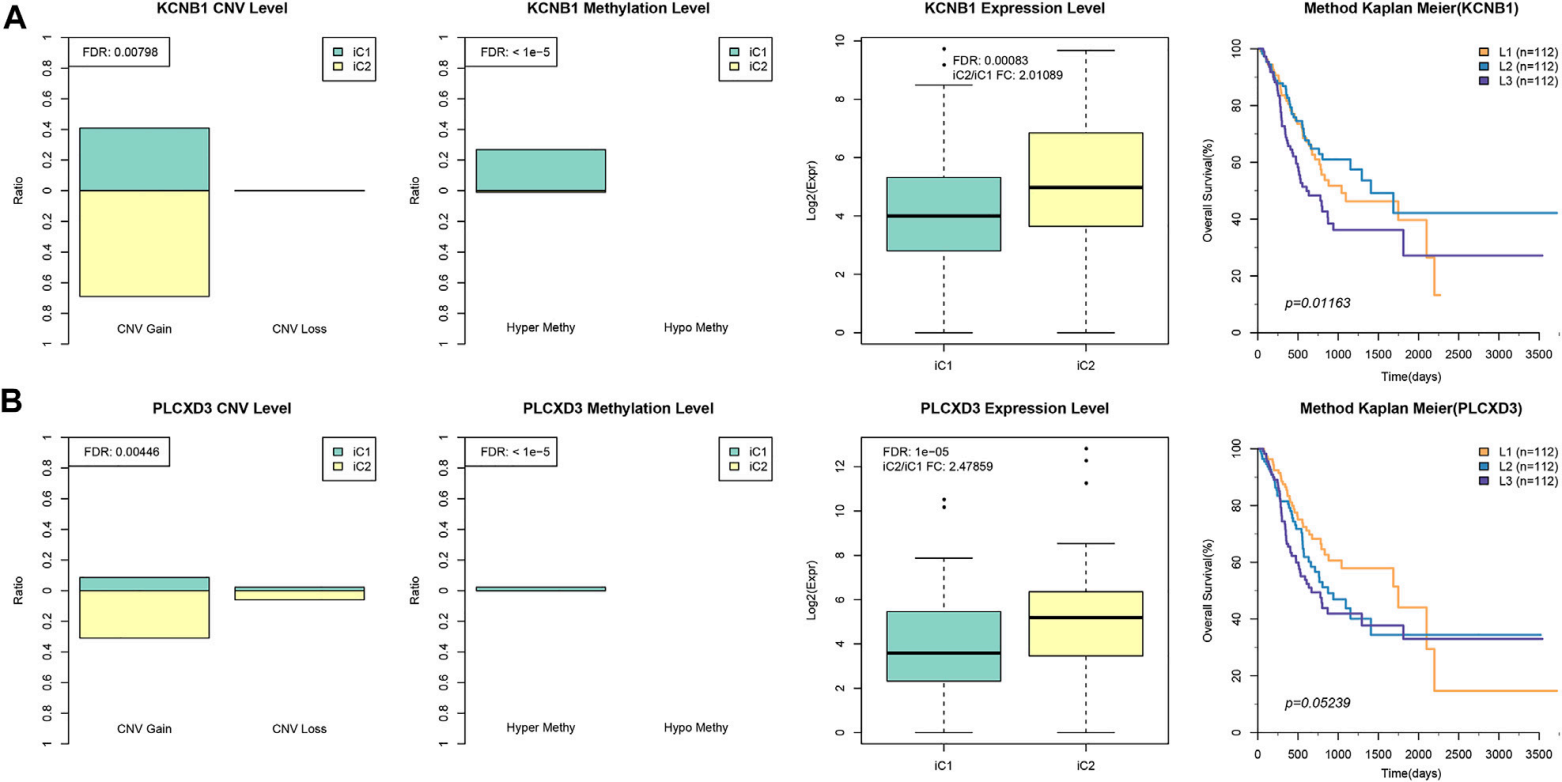
Key STAD molecular features. **(A)** The KCNB1 EXP or DNA CNV or DNA MET levels for iC1/iC2 subtypes are presented on the left. The gene expression of KCNB1 was divided into low, moderate, and high groups (L1–L3), and Kaplan–Meier curves for L1–L3 groups are displayed on the right. **(B)** The LCXD3 EXP or DNA CNV or DNA MET levels for iC1/iC3 subtypes are presented on the left, and the Kaplan–Meier curves for L1–L3 groups are displayed on the right.

Besides, differences in gene EXP, DNA CNVs, and MET between samples of iC2 and iC1 subtypes were compared. Later, DNA MET and CNV levels were classified as three types, including Normal, Gain, and Loss (for CNV), as well as Normal, HyperMethy and HypoMethy (for MET). DNA CNVcor or METcor genes with marked difference between iC2 and iC1 subtypes were obtained through Fisher’s exact test. These findings can be observed from Supplementary Tables S9 and S10. As for EXP patterns, we acquired DEGs of iC1 *versus* iC2 subtypes through DESeq2 (Love et al., 2014) (*p* < 0.05), and the results are presented in Supplementary Table S11. In addition, to illustrate the crucial prognostic features across various subtypes, altogether five genes with distinct difference between iC1 and iC2 samples at all the three (MET, CNV, and EXP) levels were screened to carry out univariate survival analysis. According to our findings, two genes (PLCXD3 and KCNB1) were related to overall survival (OS) (log-rank *p* < 0.05), suggesting that the two aforementioned genes in iC2 subtype (with poorer prognosis) had greater CNV and hypermethylation levels than iC1 subtype (with positive prognosis), and their levels within iC1 subtype were downregulated compared with those in the iC2 subtype. Afterwards, PLCXD3 and KCNB1 expression was divided as low, moderate, and high (L1–L3). Our findings suggested that L1–L3 groups with regard to PLCXD3 and KCNB1 expression levels showed positive correlation with OS (Figures 7A,B). The aforementioned findings indicated that the distinct PLCXD3 and KCNB1 expression was related to DNA MET or CNV level as well as the patient prognostic outcome. Moreover, the GEO GSE62254 (Cristescu et al., 2015) STAD dataset (*n* = 266) was utilized for analyzing the relation of those five genes with patient prognosis. The expression levels of JPH3 and KCNB1 were available, suggesting that only JPH3 in GSE62254 STAD dataset (Oh et al., 2018) showed marked correlation with prognosis. Supplementary Figure S6 displays the results. The relationship between JPH3 expression and sample OS in multiple GEO cohorts was also measured (http://kmplot.com/analysis/, Supplementary Figure S7). In addition, we determined the relationships of JPH3, PLCXD3, and KCNB1 levels with infiltrating degrees of immune cells within TME. As shown in Supplementary Figure S8, these three genes showed significant positive correlation with enrichment levels of most immune cells (CD4^+^ T, CD8^+^ T, macrophages, DCs, neutrophils).

**FIGURE 8:**
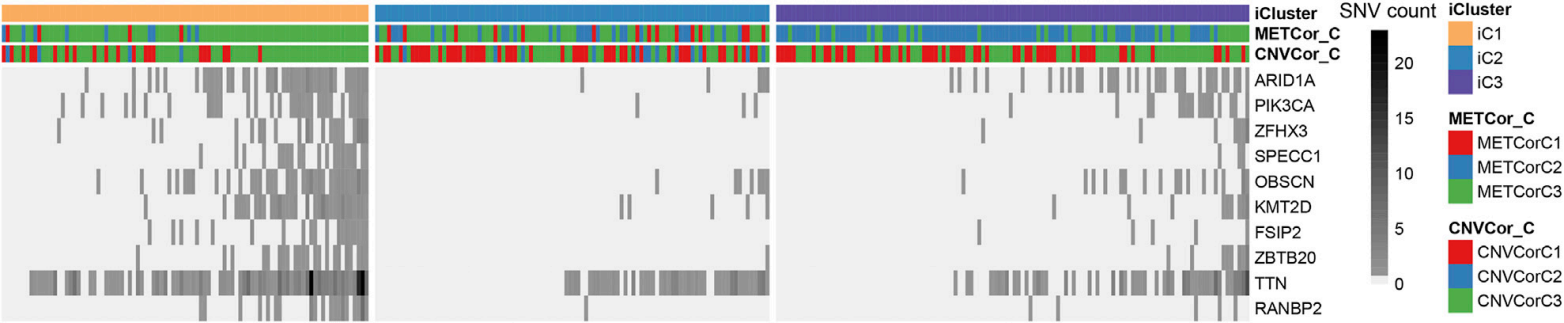
Differentially mutated genes across various STAD subtypes.

Eventually, the STAD mutation profiles were determined for exploring the associations with the sub-classification. Specifically, the synonymous mutations were eliminated to obtain the missense and nonsense mutations. Overall, the mutation frequency of genes in different subtypes showed statistically significant differences. In addition, some distinctly different mutant genes (ARID1A, PIK3CA, ZFHX3, SPECC1, OBSCN, KMT2D, FSIP2, ZBTB20, TTN, RANBP2) between iC1 and iC2 subtype were selected upon Fisher’s test, as presented in Figure 8 and Supplementary Table S12 (FDR < 0, one upon Fisher’s test). The different mutational spectra were analyzed, which suggested that the 10 genes in iC1 subtype had remarkably increased mutation frequencies compared with those in iC2 and iC3 subtypes (*p* < 0.01). Interestingly, mutation frequencies of these 10 genes in iC3 subtype with common prognosis also increased relative to iC2 subtype (*p* < 0.05). Collectively, the aforementioned findings indicated that the STAD molecular subtypes related to DNA copy numbers and DNA MET showed correlation with mutations in the 10 genes, and they might modulate the progression of STAD subtypes.

## Discussion

Prior studies indicate that comprehensive analysis on tumor genomic characteristics at different levels contributes to identifying various molecular subtypes, which can thereby shed new light on tumor clinical and mechanical differences, and reveal the potential biomarkers and therapeutic targets (Woo et al., 2017). Nonetheless, illustrating the complex STAD genomic data is still challenging. In this study, CNVcor and METcor gene dysregulation genomically and epigenetically was determined through the TCGA database. It is a strategy to integrate genomic and epigenomic data at different levels (Yeoh and Tan, 2021). According to our results, the aforementioned correlated genes contributed to identifying STAD subtypes, which reflected different molecular characteristics and genes correlated with immunity at different aspects, and prognostic outcomes. In addition, STADs that had increased frequency of abnormal CNVcor were associated with increased aberrant METcor frequency, which indicated the increased aberrant DNA MET frequency among cases showing DNA CNV frequency. The aforementioned results indicated that it was necessary to consider the aberrant DNA CNVs and MET during data analysis.

Fortunately, the current comprehensive analysis using CNVcor and METcor genes identified the new critical molecular features indicating novel STAD biomarkers and therapeutic targets. Analysis results of mutation types across different subtypes indicated that the differences in these 10 genes were significant in terms of the mutation frequency. Particularly, genes in iC1 subtype with optimal prognosis were associated with the gene mutation frequency. Five of the above 10 genes were reported to participate in STAD progression, pathogenesis, immune microenvironment, and malignant transformation, which were PIK3CA, SPECC1, ARID1A, ZBTB20, and KMT2D (Wang et al., 2011; Shi et al., 2017; Xiong et al., 2018; Ashizawa et al., 2019; Chen et al., 2019; Seo et al., 2019), and were related to the prognosis and survival for patients. Based on the aforementioned results, the bioinformatic mining results were highly reliable and accurate. Nonetheless, the associations of the five other genes with STAD have not been verified in fundamental or clinical study, which is the point that we are interested in. Among these five genes, ZFHX3 and RANBP2 are associated with an alternative mutation frequency among various malignant tumors (such as endometrial cancer and prostate cancer) (Packham et al., 2015; Walker et al., 2015; Hu et al., 2019). Nonetheless, it is still unknown about the underlying mechanism for modulating cancer genesis and development. Besides, the association of gene expression and mutation frequencies with STAD remains unknown. In this study, our results indicated that mutations in RANBP2 and PIK3CA partially mediated the favorable prognosis of STAD patients in iC1 subtype. Nonetheless, further protein–protein interaction analysis and molecular biological experiments are needed to verify these results.

Furthermore, JPH3, PLCXD3, and KCNB1 are the possible crucial regulating factors in the initiation and development of STAD. KCNB1 (Kv2.1), a major voltage-gated potassium channel (Kv), is recognized to be the new prognostic factor to predict the survival for some cancers, including glioma, and it plays a tumor-suppressing role *via* inducing autophagy (Wang et al., 2016; Wang et al., 2017). JPH3 belongs to a member of junctional membrane complex (JMC) protein family, which can stabilize the JMC between plasma membrane and endoplasmic reticulum, and maintaining the cellular ultrastructure between intracellular ionic channels and cell surface. JPH3 is recognized as the new tumor suppressor gene with methylation within colorectal cancer, thus enhancing apoptosis mediated by mitochondria. In addition, it is the candidate biomarker for survival and metastasis for digestive tract cancers (Coleman et al., 2015; Hu et al., 2017). Yet, further studies are needed to illustrate the role of PLCXD3 in STAD. Our results suggested that expression of JPH3, PLCXD3, and KCNB1 showed marked correlation with DNA MET.

## Conclusion

To sum up, the current comprehensive analysis based on gene expression at genomic and epigenomic levels reveals coordinated STAD genomic alternations at distinct points of view. Our results facilitate to determine the STAD molecular subtypes and to reveal accurate STAD clinical and mechanical diagnosis and treatments.

## Data Availability

All data utilized in this work can be accessed from the corresponding author upon request. Source code is available at: https://github.com/code1234554321/code.git

## Supplementary Material

Supplementary Figure S1 20 cluster iterations at K = 2 (category 3) were conducted for assessing iCluster cluster findings.

Supplementary Figure S2 20 cluster iterations at K = 3 (category 4) were carried out for assessing the iCluster cluster findings.

Supplementary Figure S3 Expression profiles of iCluster-identified subtypes. CNVcor **(A)** or METcor **(B)** gene–identified subtypes by NMF cluster approach are denoted in colored bars.

Supplementary Figure S4 Comparison of overall survival of STAD patients in iC3 and iC1 **(A)** or iC2 **(B)** subtypes.

Supplementary Figure S5 T **(A)**, N **(B)**, M **(C)**, stage **(D)**, grade **(E)**, and age **(F)** of STAD patients across the 3 subtypes were compared.

Supplementary Figure S6 The KCNB1 **(A)** or JPH3 **(B)** levels in GSE62254 are classified as low and high groups (L1–L2), and the Kaplan–Meier curves for L1 and L2 groups are displayed.

Supplementary Figure S7 The gene expression levels of JPH3 in other GEO cohorts are classified as low and high groups, and Kaplan–Meier curves for these two groups are displayed.

Supplementary Figure S8 The relationship between KCNB1 **(A)**, PLCXD3 **(B)**, and JPH3 **(C)** levels and infiltrating degrees of immune cells within TME.

Supplementary Table S1 Basic clinical information of STAD samples in TCGA.

Supplementary Table S2 Basic information of CNVcor genes.

Supplementary Table S3 Basic information of METcor genes.

Supplementary Table S4 CNVcor gene distribution in diverse chromosomes.

Supplementary Table S5 METcor gene distribution in diverse chromosomes.

Supplementary Table S6 Sample distribution in different subtypes discovered from iCluster analysis or METcor and CNVcor genes.

Supplementary Table S7 The levels of DNA copy-number gain/loss together with DNA hypermethylation/hypomethylation of samples.

Supplementary Table S8 Immune scores for STAD samples.

Supplementary Table S9 CNVcor genes with marked difference between iC2 and iC1 subtypes were obtained through Fisher’s exact test.

Supplementary Table S10 METcor genes with marked difference between iC2 and iC1 subtypes were obtained through Fisher’s exact test.

Supplementary Table S11 DEGs of iC1/iC2 subtypes were acquired through DESeq2.

Supplementary Table S12 Distinctly different mutant genes between iC1 and iC2 subtype were selected upon Fisher’s test.

## Abbreviations

CNV, copy number variation; CNVcor, CNVs-correlated; GC, gastric cancer; ER, endoplasmic reticulum; JMC, junctional membrane complex; MET, methylation; METcor, MET-correlated; mRNA, messenger RNA; NMF, negative matrix factorization; PM, plasma membrane; TNM, tumor node metastasis; STAD, stomach adenocarcinoma.
