# Supplementary figures and images for: Comprehensive Genomic and Epigenomic Analyses on Transcriptomic Regulation in Stomach Adenocarcinoma
Source: Front Genet. 2022 Feb 11;12:778095. doi: 10.3389/fgene.2021.778095 (PMC8873582; doi:10.3389/fgene.2021.778095)

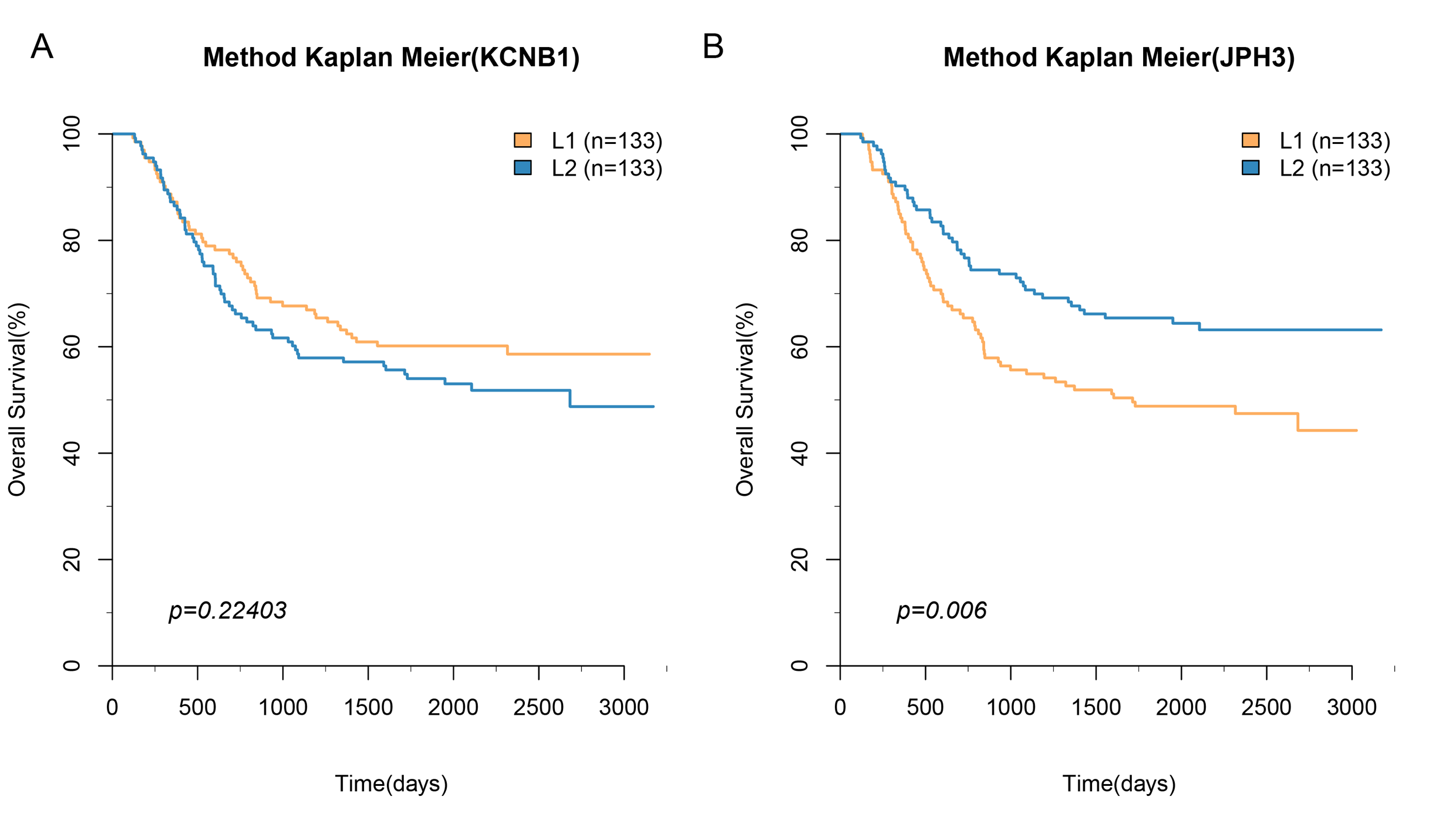

Supplement: Supplementary file 3 [file Image6.TIF]

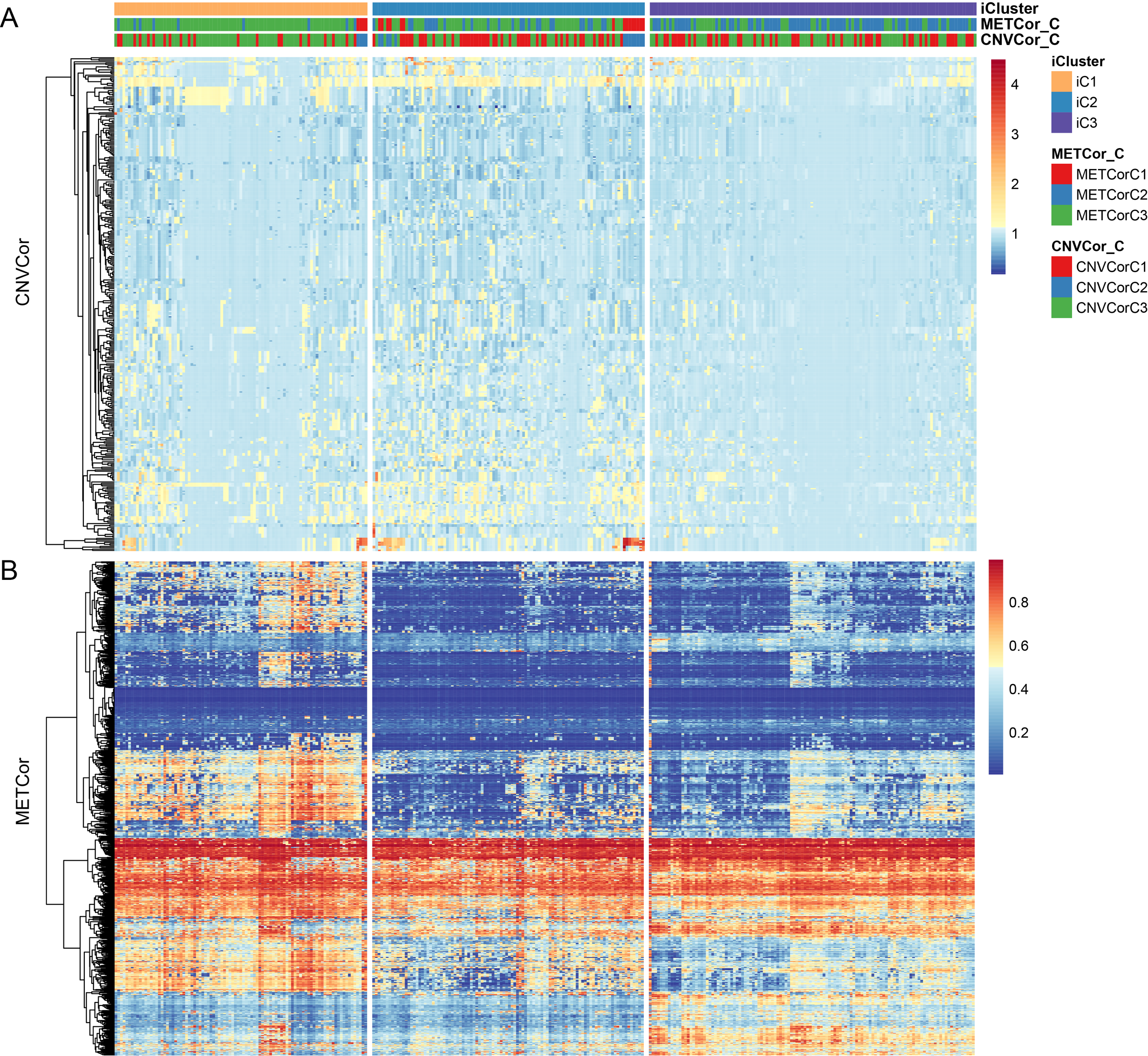

Supplement: Supplementary file 5 [file Image3.TIF]

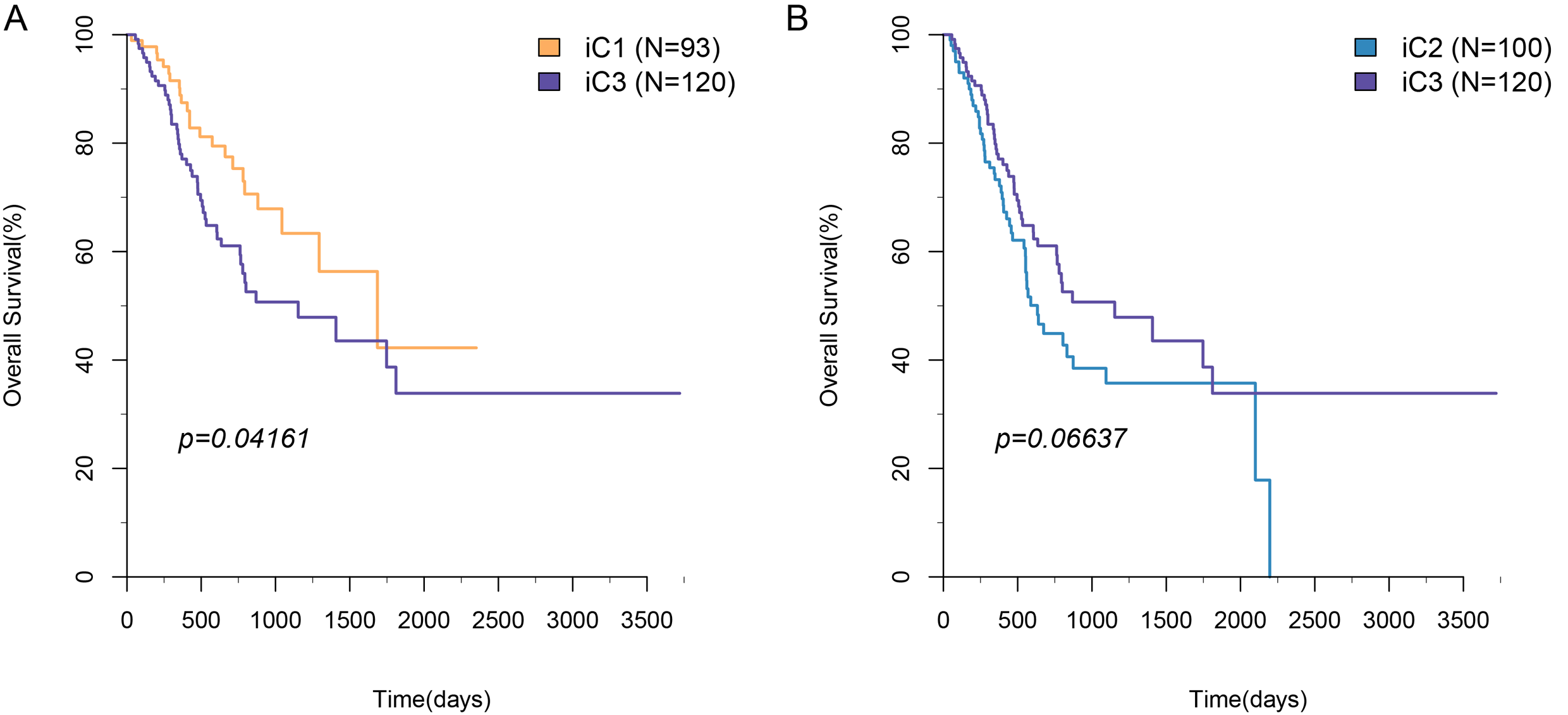

Supplement: Supplementary file 6 [file Image4.TIF]

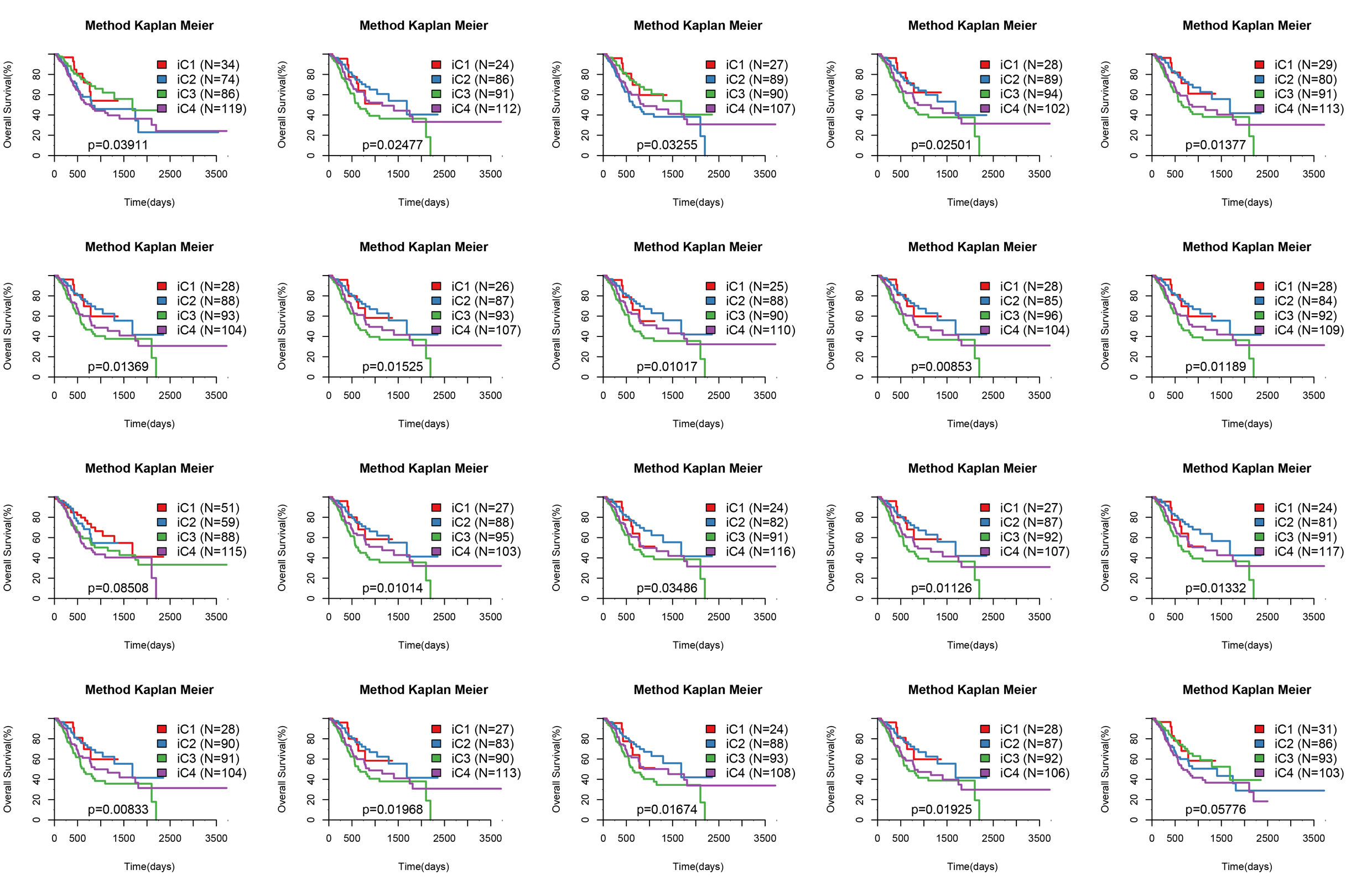

Supplement: Supplementary file 7 [file Image2.TIF]

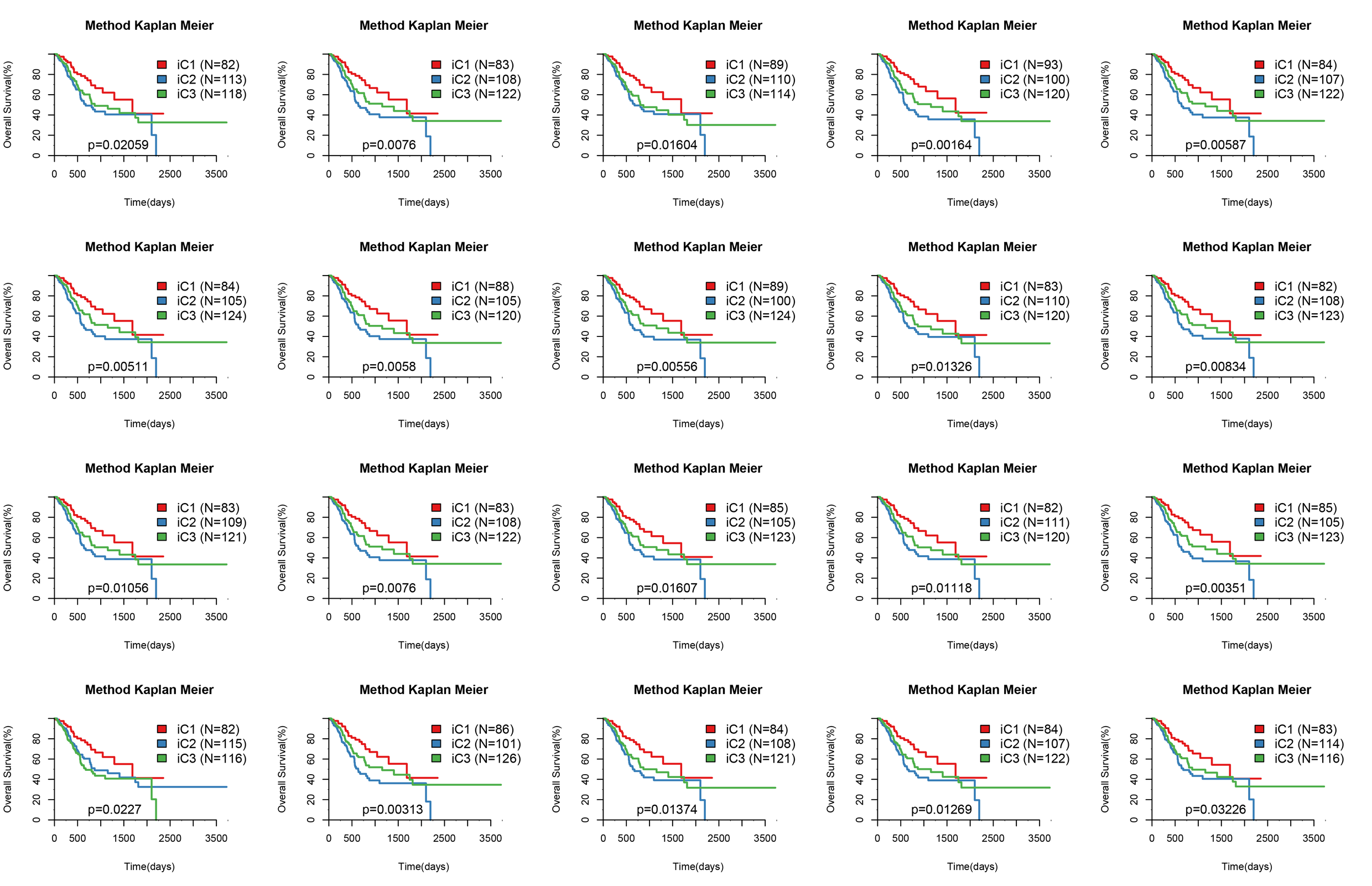

Supplement: Supplementary file 8 [file Image1.TIF]

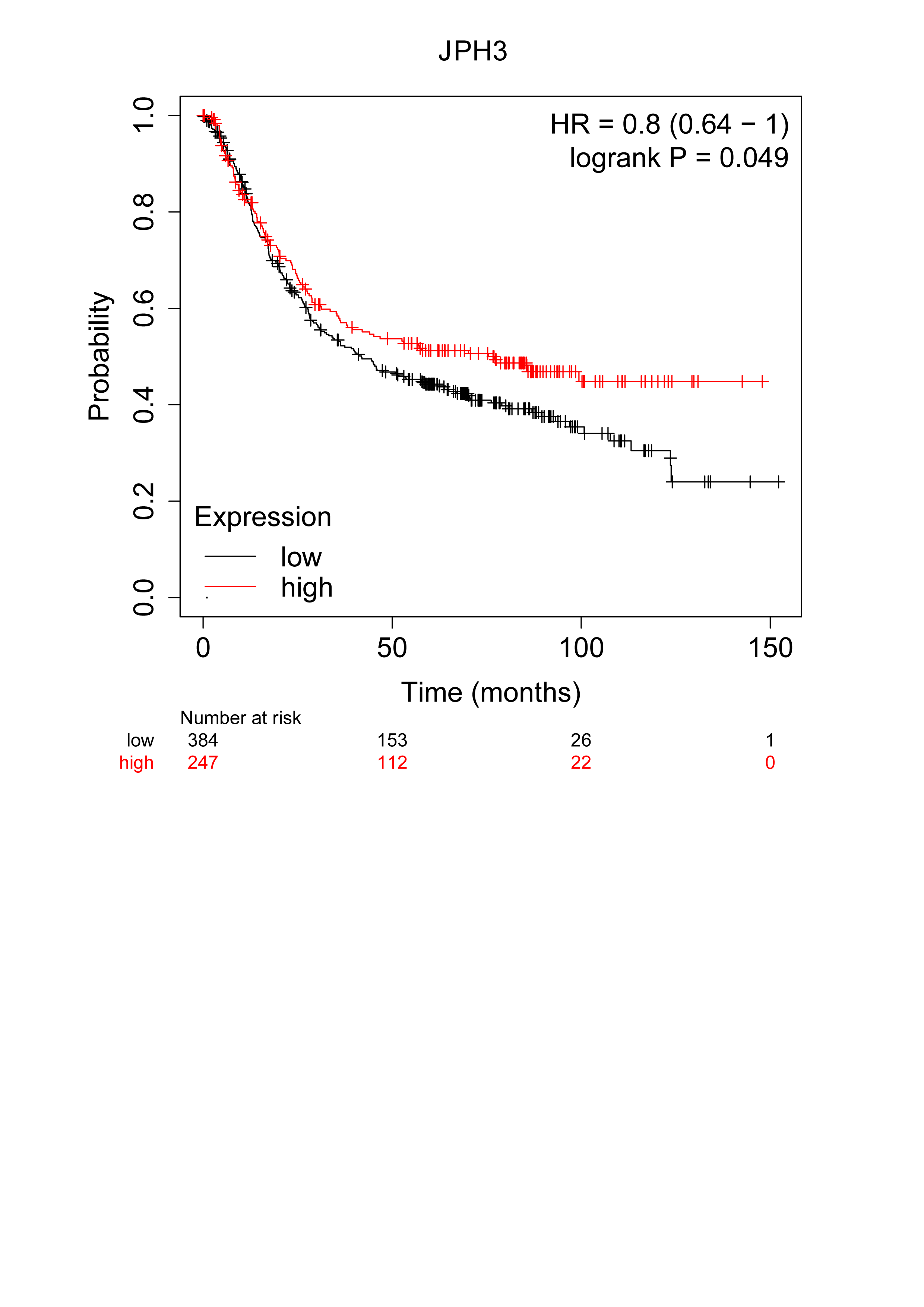

Supplement: Supplementary file 9 [file Image7.TIF]

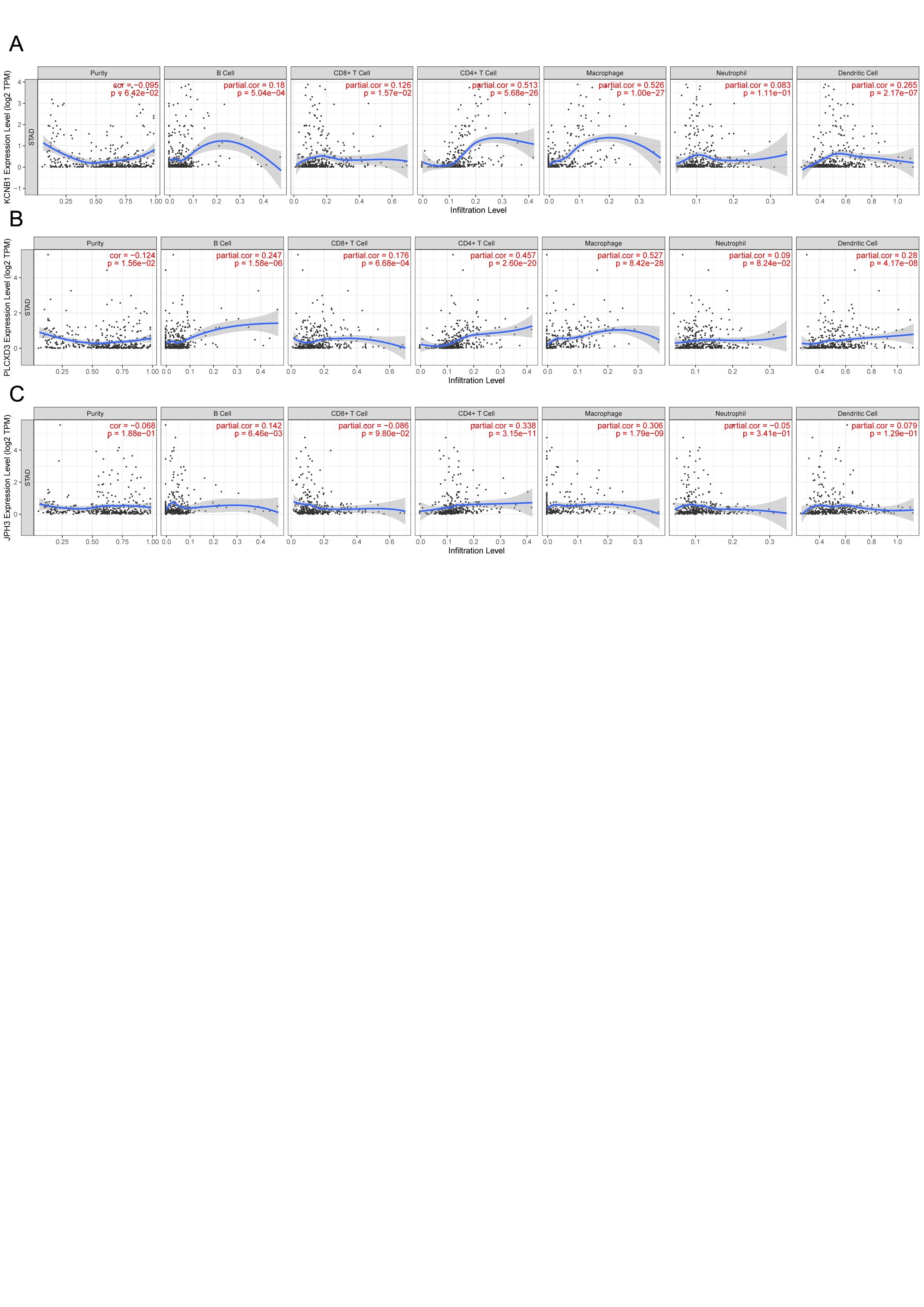

Supplement: Supplementary file 14 [file Image8.TIF]

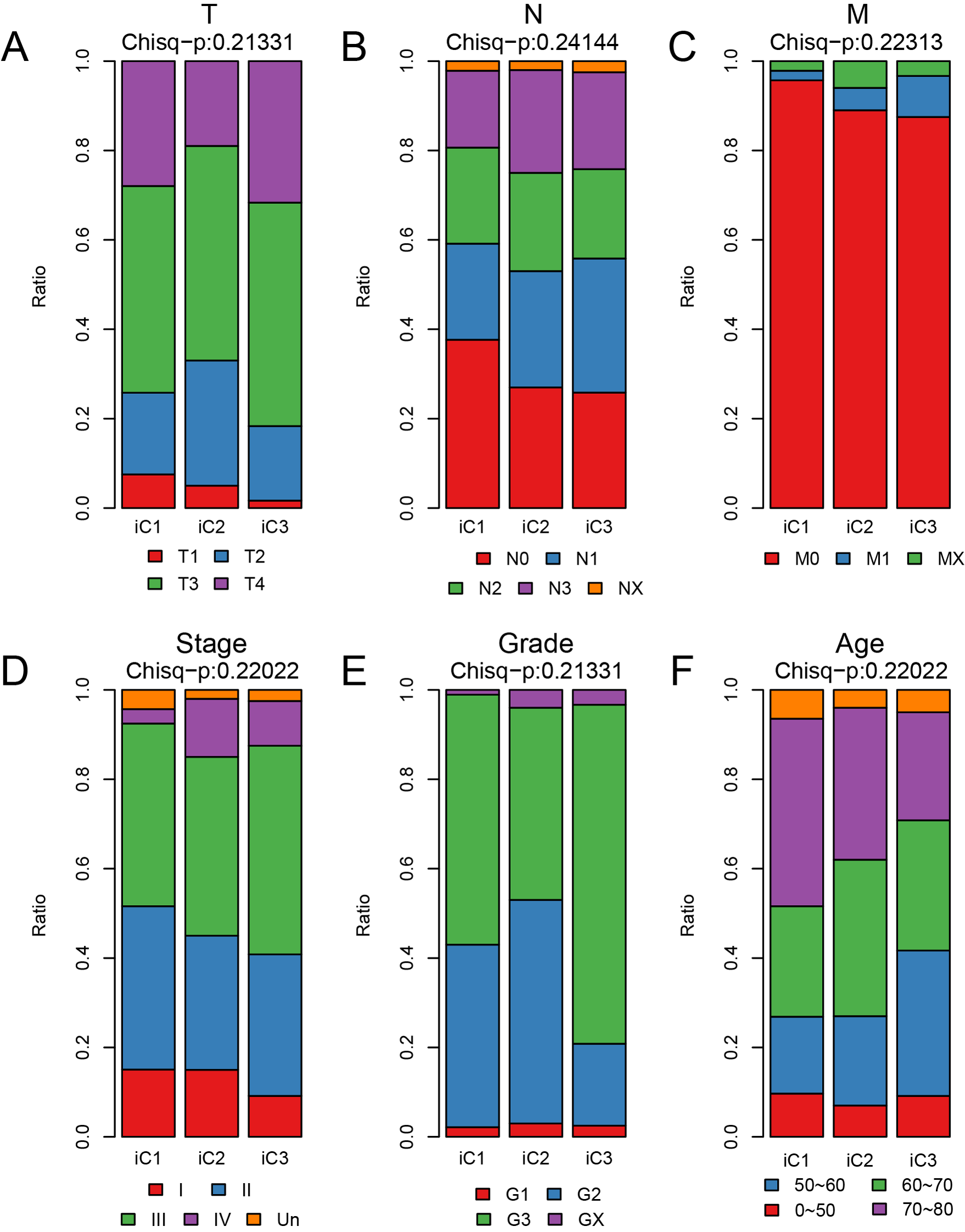

Supplement: Supplementary file 17 [file Image5.TIF]
